# The mitochondrial proteomic changes of rat hippocampus induced by 28-day simulated microgravity

**DOI:** 10.1371/journal.pone.0265108

**Published:** 2022-03-10

**Authors:** Guohua Ji, Hui Chang, Mingsi Yang, Hailong Chen, Tingmei Wang, Xu Liu, Ke Lv, Yinghui Li, Bo Song, Lina Qu

**Affiliations:** 1 State Key Laboratory of Space Medicine Fundamentals and Application, China Astronaut Research and Training Center, Beijing, China; 2 Department of Pathology and Forensics, College of Basic Medical Sciences, Dalian Medical University, Dalian, China; Universita degli studi della Campania, ITALY

## Abstract

A large number of aerospace practices have confirmed that the aerospace microgravity environment can lead to cognitive function decline. Mitochondria are the most important energy metabolism organelles, and some studies demonstrate that the areospace microgravity environment can cause mitochondrial dysfunction. However, the relationships between cognitive function decline and mitochondrial dysfunction in the microgravity environment have not been elucidated. In this study, we simulated the microgravity environment in the Sprague-Dawley (SD) rats by -30° tail suspension for 28 days. We then investigated the changes of mitochondrial morphology and proteomics in the hippocampus. The electron microscopy results showed that the 28-day tail suspension increased the mitochondria number and size of rat hippocampal neuronal soma. Using TMT-based proteomics analysis, we identified 163 differentially expressed proteins (DEPs) between tail suspension and control samples, and among them, 128 proteins were upregulated and 35 proteins were downregulated. Functional and network analyses of the DEPs indicated that several of mitochondrial metabolic processes including the tricarboxylic acid (TCA) cycle were altered by simulating microgravity (SM). We verified 3 upregulated proteins, aconitate hydratase (ACO2), dihydrolipoamide S-succinyltransferase (DLST), and citrate synthase (CS), in the TCA cycle process by western blotting and confirmed their differential expressions between tail suspension and control samples. Taken together, our results demonstrate that 28-day tail suspension can cause changes in the morphology and metabolic function of hippocampus mitochondria, which might represent a mechanism of cognitive disorder caused by aerospace microgravity.

## Introduction

Long-term space travel would adversely affect human physiology, and the most common detrimental effects include the visually impaired intracranial pressure syndrome, decreased bone density, muscle atrophy, brain functional and structural changes [1, 2]. Since spaceflight missions are rare, the ground based analogues have been developed to simulate the space environment. Animal tail suspension is a classical and useful approach for the microgravity study on Earth for a long time [3–5]. There have been several reports about the impacts of simulated microgravity (SM) on brain cognitive function. Lin et al. demonstrated that SM inhibits the proliferation of adult hippocampal neural stem cells in rats, which maybe the reason of detrimental effects of SM on learning and memory [6]. Nday et al. summarized the recently published articles and concluded that the effects of SM on brain include brain plasticity, brain neurotrophic factor (GDNF), apoptosis factors (Bcl-xL and Bax), 5-hydroxytryptamine and dopaminergic system, and dopaminergic gene expression. The neuropathological characteristics of animal SM model can be comparable to the effects of aging, anxiety and other neurological diseases [7]. Our previous research also found that tail suspension can damage the learning and memory ability of rats, and the molecules involved in glutamate excitotoxicity and several neurotransmitters (5-hydroxytryptamine, dopamine, γ-amino acid butyric acid and epinephrine) are downregulated [5, 8].

Mitochondria are essential for aerobic eukaryotes and are the most important energy supply organelles [9, 10]. Mitochondria participate in key central metabolic pathways, and are fully integrated into intracellular signaling networks that regulate multiple cellular functions, including ATP production, regulation of excitotoxicity, intracellular Ca^2+^ homeostasis, production of reactive oxygen species, release of cytochrome c and induction of cell apoptosis [11, 12]. The human mitochondrial genome (mtDNA) contains only 37 genes that encode 13 proteins [13]. The remaining thousands of mitochondrial proteins are encoded by the nuclear genome. Therefore, compared with the mtDNA, the mitochondrial proteome can provide a more comprehensive perspective for understanding mitochondrial functions. Multi-omics analysis of the hundreds of data from spaceflight for astronauts and rodents revealed that the mitochondrial processes as well as innate immunity, chronic inflammation, cell cycle, circadian rhythm, and olfactory functions are the most significant enrichment processes, and mitochondrial stress is the central biological hub of the impact of spaceflight on human beings [14]. However, the effect of SM on the hippocampus mitochondrial proteome has not been explored.

The mitochondrial dysfunction has also been demonstrated to participate in several diseases such as Alzheimer’s disease [15], cancers [16], cardiovascular diseases [17], Parkinson disease [18], traumatic brain injury and epilepsy [19, 20] etc. Therefore, the study on the mechanism of mitochondrial dysfunction would be benefit for astronauts as well as the health of people on Earth. Herein, we examined the changes in the rat hippocampus mitochondrial proteome caused by the 28-day tail suspension, and performed Gene Ontology (GO) classification, pathway enrichment and protein-protein interaction analysis on the differentially expressed proteins (DEPs). Our research can provide inspiration for understanding the mitochondrial-related molecular mechanism of cognitive function decline in microgravity.

## Materials and methods

### Animals and SM model construction

Eight weeks male Sprague-Dawley (SD) rats of SPF grade were purchased from Beijing Vital River Laboratory Animal Technology Co. Ltd., China. All rats were kept in separate cages and placed in a temperature controlled environment. The light dark cycle was 12/12 hours, and they had free access to food and water. All experimental procedures were approved by the Animal Care and Use Committee of China Astronaut Research and Training Center. After 2 weeks of adaptive feeding, the rats were randomly divided into two groups, Control group (C) and SM group (T), and each group contained 24 rats. The method of tail suspension to SM effect was described previously [21], and the Control group was raised in the identical cages without tail suspension. After 28 days of tail suspension, the animals were anesthetized by intraperitoneal injection of 10% chloral hydrate (2.5 ml/kg) and sacrificed by cervical dislocation. All efforts were made to minimize the discomfort of the animals.

### Transmission electron microscopy

After 28 days of tail suspension, the rat was euthanized by cervical dislocation and the brain was removed. The hippocampus tissue was separated on ice and cut into approximately 1mm^3^ tissue pieces, which were quickly placed in 2.5% glutaraldehyde fixative solution and fixed at 4°C overnight. Samples were rinsed three times with phosphate buffer (0.1M, pH 7.0), then fixed with 1% osmium acid solution for 2h, rinsed again with phosphate buffer for three times, and dehydrated with gradient ethanol, put in epoxy resin, treated at 80°C for 24h to polymerize, then cut into 100nm ultra-thin sections and dyed with uranyl acetate and lead citrate. Finally it was observed under a transmission electron microscope (Hitachi, Japan). The number of mitochondria in each neuronal soma was counted from at least 10 cells in each group. The micrographs were tracked respectively, and the shape and size parameters were obtained by ImageJ software. The surface area representing the size of mitochondria was reported in squared micrometers (μm^2^), and Ferret’s diameter was measured by the longest distance between two points within a mitochondria.

### Isolation of mitochondria

Fifteen rats were randomly taken out from the tail suspension and control groups. After separating the hippocampus, the left and right hippocampus of five randomly selected rats were mixed together to form a sample. The mitochondria were extracted by Mitochondrial Isolation Kit (MP-007, Invent, USA) according to the manufacture’s instructions. Protein concentrations were quantified by BCA assay (Thermo Fischer, USA).

### TMT-based proteomics analysis

#### Protein processing

Dissolved the mitochondria with Protein Solubilization Reagent for MS (WA-011, Invent, USA). Added the DTT to the sample to a final concentration of 10mM and reduced in the oven at 55°C for 1h. After the samples returning to the room temperature, added the IAM at a final concentration of 40mM, and reacted for 30min in dark. Centrifuged with a 10kda ultrafiltration tube at 12000g, and added 200μl of 100mM TEAB to the samples, then added trypsin according to 1/50 of the protein mass, and kept in a water bath at 37°C overnight. The next day, washed 3 times with ultrapure water and freeze-dried at the bottom of the enrichment tube.

#### TMT labelling

Re-dissolved the peptides with 200μl of 200mM TEAB, and then quantified them with NanoDrop (Thermo Fisher, USA). Taking 25μg of peptides from each sample and labelled them with TMT reagent at room temperature for 1 hour. The labeled samples were as follows: the Control group, labeled with 126, 127N and 128N; the SM group, labeled with 129N, 130N and 131. Then added 5μl of 5% ammonia water for quenching, mixed and reacted at room temperature for 15 minutes, and then mixed 5 labeled samples from same group together and evaporated to dryness at 60°C by a concentrator.

#### HPLC liquid phase separation

Prepared mobile phase A liquid (2% acetonitrile, 98% water, ammonia water adjusted to pH = 10) and B liquid (98% acetonitrile, 2% water, ammonia water adjusted to pH = 10). Used 150μl of solution A to dissolve the lyophilized powder of the sample, centrifuged at 12000g for 10min at room temperature, and took the supernatant for injection. The TMT labelled peptide mixture was fractionated by a Durashell column from Agela (4.6mm×250mm i.d, C18, 5μm) by L-3000 HPLC system (Rigol, China). A total of 100 tubes were collected with the speed of 1 tube per minute, and finally combined into 10 fractions. All fractions were dried by a rotary vacuum concentrator.

#### LC-MS/MS analysis

The TMT-labeled sample of each fraction was resuspended in 2% acetonitrile, 98% water and 0.1% FA, after centrifuged at 12000g for 3min, 10μl of supernatant was loaded onto an Eksigent Nano LC 2D plus HPLC by the autosampler onto a 5μm C18 trap column (ID100μm, 20mm length). The peptides were then eluted onto a 3μm analytical C18 column (ID75μm, 120mm length) packed in-house. Separation was run at 330 nl/min starting from 5% B2 (98% ACN+1.9% H2O+0.1% FA), followed by stepwise gradient (8% B2 for 5min, 22% B2 for 34min, 32% B2 for 41min, 90% B2 for 42min), maintained at 90% B2 for another 46min, and finally returned to 5% B2 for 1min. LC-MS/MS analysis was performed on a Q Exactive HF mass spectrometer (Thermo Fisher, USA). The mass spectrometry parameters were set as follows: the first-level M/Z scan range was 300–1400, the resolution was 120,000, the AGC target was 3e6, the maximum ion injection time was 80ms, 15 precursor ions were selected for secondary fragmentation, and the secondary resolution was 60000. The AGC target was 5e4, the maximum ion injection time was 20ms, and the precursor ion window was set to 1.2M/Z. Proteome Discoverer (version 2.3.1.15, Thermo Fisher Scientific, USA) was used for data retrieval of raw data. The database used was downloaded from uniprotKB (including TrEMBL entries) on August 13, 2020, containing 20289 sequence information, and was also downloaded from Uniprot_Rat (version 2019.04.20). Andromeda search engine was used with the following settings: trypsin cleavage; fixed modification of carbamidomethylation of cysteine; variable modifications of oxidation of methionine; acetylation modification at the N-terminal of protein, the primary mass error was set at 20ppm and the secondary mass error was set at 20mmu; a maximum of two missed cleavage. The false discovery rate was calculated by decoy database searching. For protein identification, the peptides were of minimum 6 amino acids and had at least 1 unique peptide identified per protein. A false discovery rate (FDR) of 1% at both peptide and protein level was used. Normalization was performed against the total peptide amount.

### Analysis of mitochondrial proteome

In order to determine the possible biological functions of DEPs, DAVID Bioinformatics Resources were used to conduct gene ontology (GO) analysis in biological processes, cell components and molecular functions. The Kyoto Encyclopedia of Genes and Genomes (KEGG: http://www.genome.jp/kegg/) analysis was also conducted. The protein-protein interaction (PPI) network was constructed using the STRING database and Cytoscape.

### Western blotting

Mitochondria extraction and dissolution were conducted as described above, and the protein concentration was determined with BCA Protein Assay Kit (Thermo Fisher, USA). Western blot analyses were performed according to standard procedures. The primary antibodies used were aconitate hydratase (ACO2, 6571), dihydrolipoamide S-succinyltransferase (DLST, 11954), citrate synthase (CS, 14309), and VDAC (4661), and all of the antibodies were purchased from Cell Signaling Technology, Danvers, MA, USA. The immunoblot images were analyzed with ImageJ to determine the relative integrated density, and the relative expressions of ACO2, DLST and CS were represented by the intensity ratio between the interested protein and the loading control (VDAC) in each loading lane.

### Statistical analysis

The data were expressed as mean±standard deviation (SD) or fold change (FC) relative to the corresponding control group. Statistical analysis was performed using two tailed Student’s t-test. The Fisher’s exact test was used to evaluate the significance of GO terms and Pathway enrichment, with correction for multiple comparisons based on the false discovery rate (FDR). A p-value or FDR less than 0.05 were considered statistically significant.

## Results

### Mitochondrial dynamics and morphology are changed by 28-day SM

**Fig 1:**
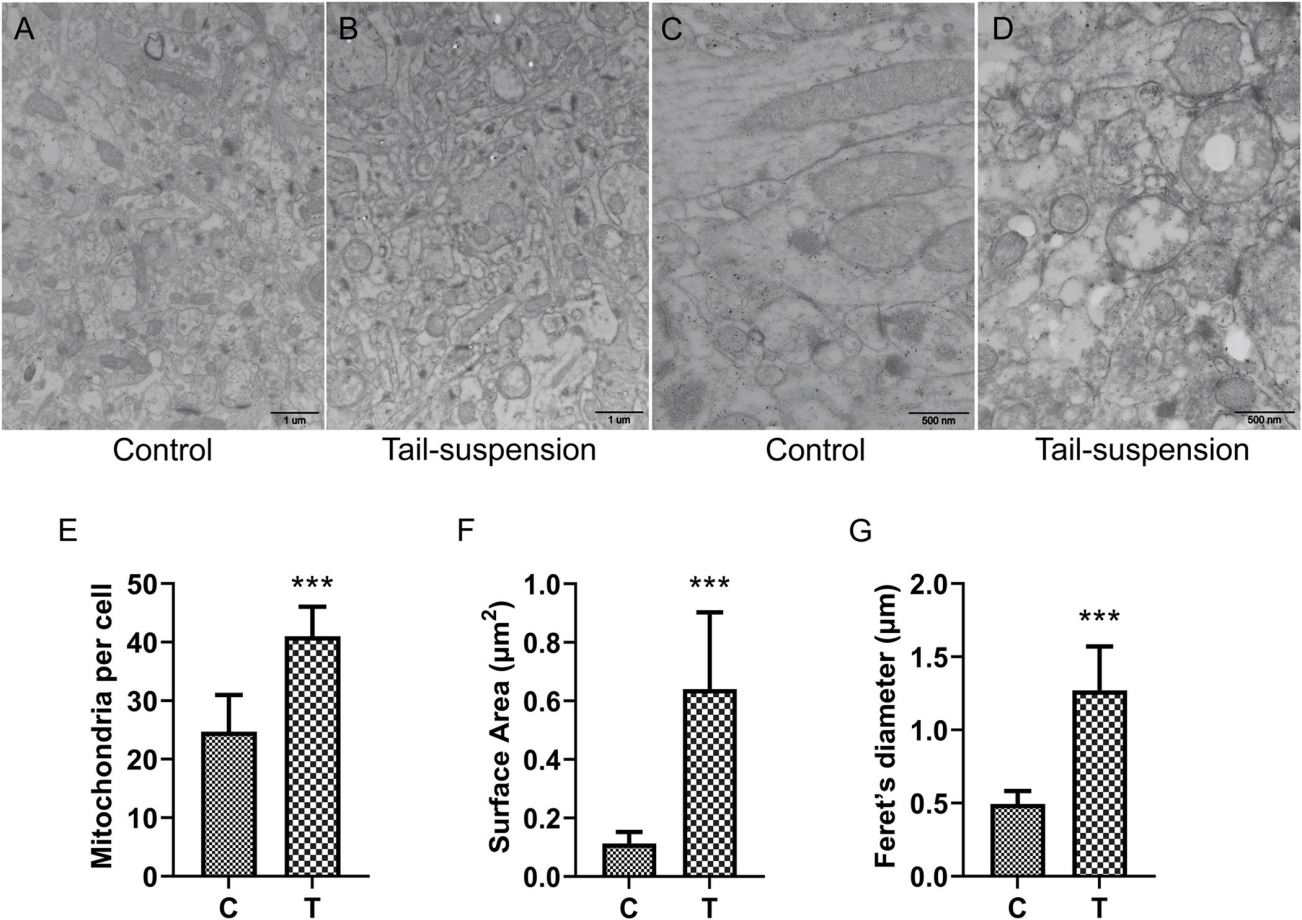
Effects of SM on mitochondrial morphology and dynamics of hippocampal neuronal soma in rats. Rats were tail suspended for 28 days, and the mitochondria in hippocampal neuronal soma were observed under transmission electron microscope. **(A, B)** Zoom 20,000 times. n = 6. **(C, D)** Zoom 50,000 times. **(E)** Mitochondria per cell were counted. n≥10 images per group. **(F, G)** mitochondrial mean surface area and Feret’s diameter on transmission electron microscopy images. n = 25. C and T represented the Control group and SM group samples respectively. ***p<0.001.

We studied the influence of SM on the dynamics and morphology of mitochondria in hippocampal neuronal soma using transmission electron microscopy. It was found that the mitochondrial swelling was obvious, and the cristae were loose and dissolved in the SM group, while the mitochondrial structure in the Control group was basically normal, the membrane shape was complete, and the cristae were dense and regular (**Fig 1A–1D**). The number of mitochondria in the SM group was significantly increased compared to the Control group (p<0.001), which was reflected by the elevated surface area and the lengthened Feret’s diameter (**Fig 1E–1G**). These data suggest that the function of mitochondria in the rat hippocampal neuronal soma is changed under the 28-day SM condition.

### The mitochondrial proteome of hippocampus is altered by SM

**Fig 2:**
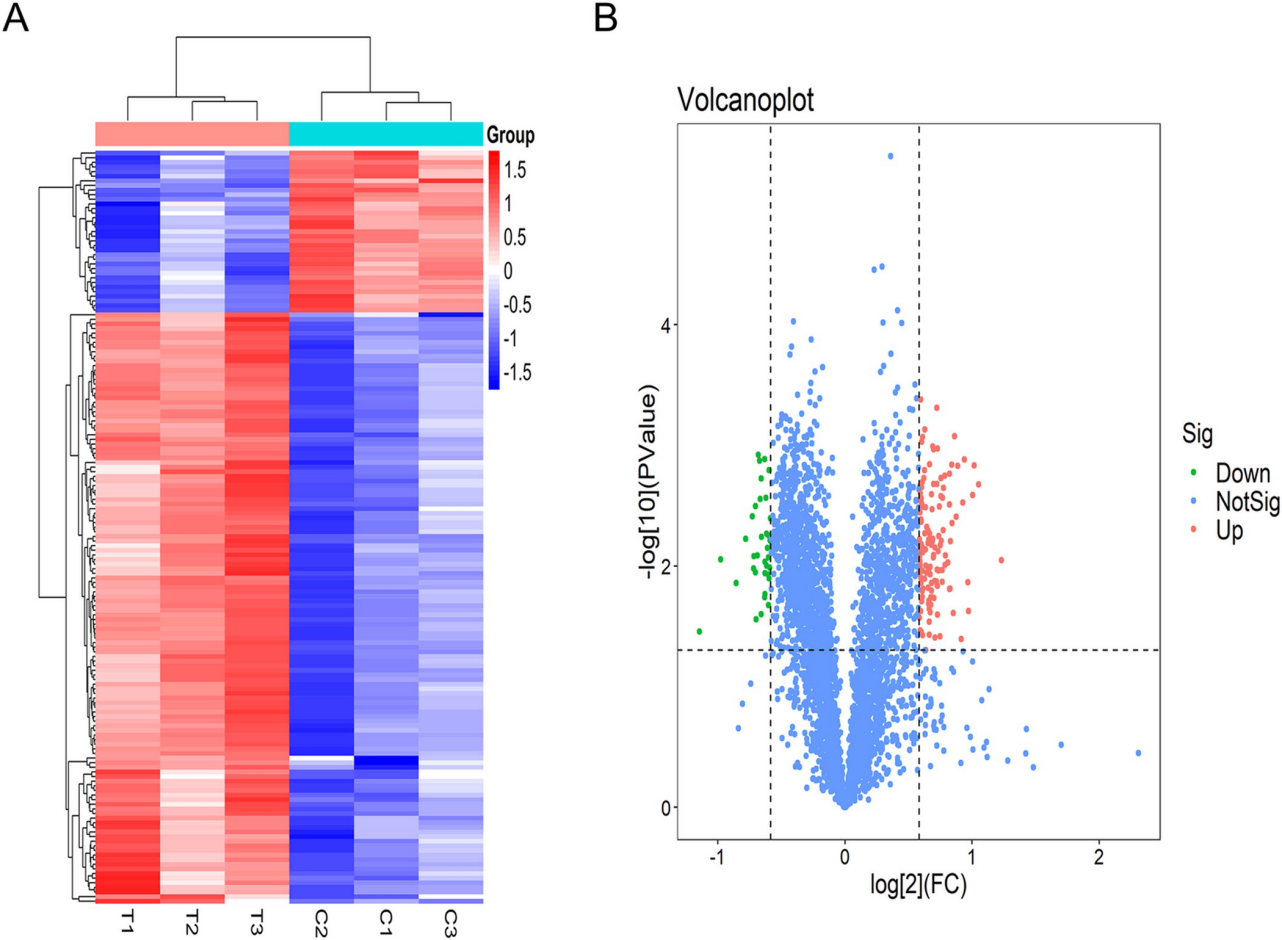
The overview of global changes of identified proteins. **(A)** The identified proteins expression profile by heatmap plot. T1, T2 and T3, and C1, C2 and C3 respectively represented 3 tail suspension samples and 3 control samples. **(B)** Volcano plots of fold-change vs. -log_10_ p-value of identified proteins. The significance threshold was set at p-value≤0.05. The significant differentially expressed proteins were marked with different colors, the upregulated proteins were marked in pink, downregulated proteins were marked in green, and blue dots indicated no significant difference. FC, fold change.

To evaluate the changes in hippocampus mitochondrial protein expression, we performed TMT-based proteomics analysis on the SM group and Control group samples. The heatmap plot disclosed that a total of 4,044 proteins were quantified across all samples, and the hippocampus mitochondrial protein expression was altered significantly by SM (**Fig 2A**). A fold change cutoff value of ≥1.5 or ≤0.67 was defined as up- or down-regulation, and only proteins that were identified by three or more peptides with >1.5-fold changes and statistically significant (p value≤0.05) were considered to be DEPs. Among the 163 DEPs, 128 proteins were upregulated in the SM group compared with the Control group, whereas 35 proteins were downregulated (**Fig 2B**). The gene and protein names, fold change and p-value of DEPs were listed in **Table 1** and **S1 Table**.

**Table 1:** The protein changes of mitochondria of hippocampus after 28 days of tail suspension in rats.

ID	Accession	Gene name	Protein name	Fold change	p-value
A0A0G2JYW6_RAT			Uncharacterized protein	2.35	0.008939
HOT_RAT	Q4QQW3	Adhfe1	Hydroxyacid-oxoacid transhydrogenase, mitochondrial	2.07	0.002111
G3V8U8_RAT	G3V8U8	Bcat2	Branched-chain-amino-acid aminotransferase	2.02	0.001467
SODM_RAT	P07895	Sod2	Superoxide dismutase [Mn], mitochondrial	2.01	0.00258
ATP6_RAT	P05504	Mt-atp6	ATP synthase subunit a	1.96	0.02367
AATM_RAT	P00507	Got2	Aspartate aminotransferase, mitochondrial	1.95	0.013689
CH10_RAT	P26772	Hspe1	10 kDa heat shock protein, mitochondrial	1.92	0.001314
GCSH_RAT	Q5I0P2	Gcsh	Glycine cleavage system H protein, mitochondrial	1.90	0.002974
TRXR2_RAT	Q9Z0J5	Txnrd2	Thioredoxin reductase 2, mitochondrial	1.88	0.040301
G3V936_RAT	G3V936	Cs	Citrate synthase	1.85	0.00148
D3ZT98_RAT	D3ZT98	Bola3	BolA family member 3	1.83	0.003921
GABT_RAT	P50554	Abat	4-aminobutyrate aminotransferase, mitochondrial	1.82	0.000839
A0A0G2K2Q2_RAT	A0A0G2K2Q2	Gcat	Glycine C-acetyltransferase	1.81	0.024635
AUHM_RAT	F1LU71	Auh	Methylglutaconyl-CoA hydratase, mitochondrial	1.79	0.004409
ES1_RAT	P56571		ES1 protein homolog, mitochondrial	1.79	0.001447
FAHD1_RAT	Q6AYQ8	Fahd1	Acylpyruvase FAHD1, mitochondrial	1.78	0.006231
G3V945_RAT	G3V945	Aldh5a1	Succinate-semialdehyde dehydrogenase	1.77	0.015407
A0A0G2JVW3_RAT	A0A0G2JVW3	Ankrd17	Ankyrin repeat domain 17	1.77	0.015563
IDHP_RAT	P56574	Idh2	Isocitrate dehydrogenase [NADP], mitochondrial	1.77	0.003087
DHE3_RAT	P10860	Glud1	Glutamate dehydrogenase 1, mitochondrial	1.77	0.001715
F1LN88_RAT	F1LN88	Aldh2	Aldehyde dehydrogenase, mitochondrial	1.75	0.00937
IVD_RAT	P12007	Ivd	Isovaleryl-CoA dehydrogenase, mitochondrial	1.73	0.008189
THTR_RAT	P24329	Tst	Thiosulfate sulfurtransferase	1.73	0.009805
ECHM_RAT	P14604	Echs1	Enoyl-CoA hydratase, mitochondrial	1.72	0.001788
ACON_RAT	Q9ER34	Aco2	Aconitate hydratase, mitochondrial	1.71	0.002251
CALR_RAT	P18418	Calr	Calreticulin	1.71	0.00505
PPIF_RAT	P29117	Ppif	Peptidyl-prolyl cis-trans isomerase F, mitochondrial	1.70	0.002718
B2RYT0_RAT	B2RYT0	Mrps21	Mitochondrial ribosomal protein S21	1.70	0.010777
D4A5F4_RAT	D4A5F4	RGD1311575	Hypothetical LOC289568	1.70	0.013594
ECH1_RAT	Q62651	Ech1	Delta(3,5)-Delta(2,4)-dienoyl-CoA isomerase, mitochondrial	1.69	0.006191
ETFA_RAT	P13803	Etfa	Electron transfer flavoprotein subunit alpha, mitochondrial	1.69	0.002644
G3V9U2_RAT	G3V9U2	Acaa2	3-ketoacyl-CoA thiolase, mitochondrial	1.69	0.001865
D4A7X5_RAT	D4A7X5	Ppm1k	Protein phosphatase 1K (PP2C domain containing) (Predicted)	1.68	0.006727
KNG1_RAT	P08934	Kng1	Kininogen-1	1.68	0.038332
G3V7I0_RAT	G3V7I0	Prdx3	Peroxiredoxin 3	1.67	0.003211
F1LNF7_RAT	F1LNF7	Idh3a	Isocitrate dehydrogenase [NAD] subunit, mitochondrial	1.67	0.005636
Q6IMX3_RAT	Q6IMX3	Acads	Acetyl-Coenzyme A dehydrogenase, short chain, isoform CRA_a	1.67	0.01089
A0A0G2JVM0_RAT	A0A0G2JVM0	Aldh4a1	Delta-1-pyrroline-5-carboxylate dehydrogenase, mitochondrial	1.66	0.007541
D4A8N2_RAT	D4A8N2	Fdx2	Ferredoxin 2	1.66	0.0146
A0A0G2JZA2_RAT	A0A0G2JZA2	Grpel1	GrpE protein homolog	1.66	0.02531
3HIDH_RAT	P29266	Hibadh	3-hydroxyisobutyrate dehydrogenase, mitochondrial	1.66	0.001062
MESD_RAT	Q5U2R7	Mesd	LRP chaperone MESD	1.65	0.010713
GATA_RAT	Q5FWT5	Qrsl1	Glutamyl-tRNA(Gln) amidotransferase subunit A, mitochondrial	1.65	0.014454
FUMH_RAT	P14408	Fh	Fumarate hydratase, mitochondrial	1.65	0.000491
A0A0G2K9G3_RAT	A0A0G2K9G3	Mrps24	Mitochondrial ribosomal protein S24	1.65	0.00209
D4ADD7_RAT	D4ADD7	Glrx5	Glutaredoxin 5	1.65	0.013459
Q6AXY8_RAT	Q6AXY8	Dhrs1	Dehydrogenase/reductase (SDR family) member 1	1.63	0.039263
MDHM_RAT	P04636	Mdh2	Malate dehydrogenase, mitochondrial	1.63	0.007061
G3V7J0_RAT	G3V7J0	Aldh6a1	Aldehyde dehydrogenase family 6, subfamily A1, isoform CRA_b	1.63	0.013342
MAAI_RAT	P57113	Gstz1	Maleylacetoacetate isomerase	1.63	0.018412
ACADL_RAT	P15650	Acadl	Long-chain specific acyl-CoA dehydrogenase, mitochondrial	1.62	0.002101
LYRM9_RAT	B2RZD7	Lyrm9	LYR motif-containing protein 9	1.62	0.001083
THIL_RAT	P17764	Acat1	Acetyl-CoA acetyltransferase, mitochondrial	1.62	0.008093
MANF_RAT	P0C5H9	Manf	Mesencephalic astrocyte-derived neurotrophic factor	1.62	0.008521
F1M5N4_RAT	F1M5N4	Me3	Malic enzyme	1.62	0.005954
D4A0Y4_RAT	D4A0Y4	Oxnad1	Oxidoreductase NAD-binding domain containing 1 (Predicted), isoform CRA_b	1.61	0.001023
A0A0A0MXW1_RAT	A0A0A0MXW1	Bckdhb	2-oxoisovalerate dehydrogenase subunit beta, mitochondrial	1.61	0.006284
G3V796_RAT	G3V796	Acadm	Acetyl-Coenzyme A dehydrogenase, medium chain	1.61	0.0034
A0A0G2K5F1_RAT	A0A0G2K5F1	Macrod1	ADP-ribose glycohydrolase MACROD1	1.61	0.018189
IDH3B_RAT	Q68FX0	Idh3B	Isocitrate dehydrogenase [NAD] subunit beta, mitochondrial	1.60	0.002549
G3V7I5_RAT	G3V7I5	Aldh1b1	Aldehyde dehydrogenase X, mitochondrial	1.60	0.006674
D3ZKG1_RAT	D3ZKG1	Mmut	Methylmalonyl CoA mutase	1.60	0.015932
F210A_RAT	Q5XIJ4	Fam210a	Protein FAM210A	1.60	0.013908
FMT_RAT	Q5I0C5	Mtfmt	Methionyl-tRNA formyltransferase, mitochondrial	1.60	0.018123
CEGT_RAT	Q9R0E0	Ugcg	Ceramide glucosyltransferase	1.60	0.020969
A0A0H2UI21_RAT	A0A0H2UI21	Crat	Carnitine O-acetyltransferase	1.59	0.011738
M0R3V4_RAT	M0R3V4	Mydgf	Myeloid-derived growth factor	1.59	0.010141
OAT_RAT	P04182	Oat	Ornithine aminotransferase, mitochondrial	1.59	0.024142
F1LPV8_RAT	F1LPV8	Suclg2	Succinate—CoA ligase [GDP-forming] subunit beta, mitochondrial	1.59	0.005411
A0A0G2JSS8_RAT	A0A0G2JSS8	Prdx5	Peroxiredoxin	1.59	0.001438
RCN2_RAT	Q62703	Rcn2	Reticulocalbin-2	1.59	0.028981
ATIF1_RAT	Q03344	Atp5if1	ATPase inhibitor, mitochondrial	1.58	0.037234
Q5U3Z7_RAT	Q5U3Z7	Shmt2	Serine hydroxymethyltransferase	1.58	0.022602
SCOT1_RAT	B2GV06	Oxct1	Succinyl-CoA:3-ketoacid coenzyme A transferase 1, mitochondrial	1.58	0.019463
Q68FZ8_RAT	Q68FZ8	Pccb	Propionyl coenzyme A carboxylase, beta polypeptide	1.58	0.01069
FAHD2_RAT	B2RYW9	Fahd2	Fumarylacetoacetate hydrolase domain-containing protein 2	1.58	0.006075
F1M8H2_RAT	F1M8H2	Wars2	Tryptophanyl tRNA synthetase 2 (mitochondrial)	1.58	0.020609
Q6AY99_RAT	Q6AY99	Akr1b10	Aldo-keto reductase family 1 member B10	1.57	0.015156
A0A0H2UHE1_RAT	A0A0H2UHE1	Suclg1	Succinate—CoA ligase [ADP/GDP-forming] subunit alpha, mitochondrial	1.57	0.005037
D4A830_RAT	D4A830	Ppa2	Pyrophosphatase (inorganic) 2	1.57	0.006482
F1LP30_RAT	F1LP30	Mccc1	Methylcrotonoyl-CoA carboxylase subunit alpha, mitochondrial	1.56	0.01137
D3ZTR1_RAT	D3ZTR1	Mrps17	Mitochondrial ribosomal protein S17	1.56	0.004775
Q5RJR9_RAT	Q5RJR9	Serpinh1	Serine (Or cysteine) proteinase inhibitor, clade H, member 1, isoform CRA_b	1.55	0.049977
G3V6T7_RAT	G3V6T7	Pdia4	Protein disulfide-isomerase A4	1.55	0.009949
C1QBP_RAT	O35796	C1qbp	Complement component 1 Q subcomponent-binding protein, mitochondrial	1.55	0.00327
D4A833_RAT	D4A833	Mrps30	Mitochondrial ribosomal protein S30	1.55	0.007282
DLDH_RAT	Q6P6R2	Dld	Dihydrolipoyl dehydrogenase, mitochondrial	1.55	0.005384
A0A0H2UI42_RAT	A0A0H2UI42	Mrpl30	39S ribosomal protein L30, mitochondrial	1.54	0.00074
AL7A1_RAT	Q64057	Aldh7a1	Alpha-aminoadipic semialdehyde dehydrogenase	1.54	0.012773
HMCS2_RAT	P22791	Hmgcs2	Hydroxymethylglutaryl-CoA synthase, mitochondrial	1.54	0.001604
DHTK1_RAT	Q4KLP0	Dhtkd1	Probable 2-oxoglutarate dehydrogenase E1 component DHKTD1, mitochondrial	1.54	0.001875
F1LM47_RAT	F1LM47	Sucla2	Succinate—CoA ligase [ADP-forming] subunit beta, mitochondrial	1.53	0.003233
M0R4L6_RAT	M0R4L6	Gatb	Glutamyl-tRNA(Gln) amidotransferase subunit B, mitochondrial	1.53	0.003219
G3V6P2_RAT	G3V6P2	Dlst	Dihydrolipoamide S-succinyltransferase (E2 component of 2-oxo-glutarate complex), isoform CRA_a	1.53	0.015357
A0A0G2JUZ5_RAT	A0A0G2JUZ5	Gldc	Glycine cleavage system P protein	1.53	0.037413
CATB_RAT	P00787	Ctsb	Cathepsin B	1.53	0.000854
G3V6F5_RAT	G3V6F5	Elac2	ElaC homolog 2 (E. coli)	1.53	0.007722
SDHF1_RAT	B0K036	Sdhaf1	Succinate dehydrogenase assembly factor 1, mitochondrial	1.53	0.016151
RM38_RAT	Q5PQN9	Mrpl38	39S ribosomal protein L38, mitochondrial	1.53	0.018071
D3ZDX7_RAT	D3ZDX7	Mrpl48	Mitochondrial ribosomal protein L48	1.53	0.007002
A0A0G2K7D7_RAT	A0A0G2K7D7	Nars2	Asparaginyl-tRNA synthetase 2, mitochondrial	1.52	0.002014
G3V879_RAT	G3V879	Coq7	5-demethoxyubiquinone hydroxylase, mitochondrial	1.52	0.010989
ETFB_RAT	Q68FU3	Etfb	Electron transfer flavoprotein subunit beta	1.52	0.003036
D3ZDP2_RAT	D3ZDP2	Mrpl58	Mitochondrial ribosomal protein L58	1.52	0.01954
G3V8W9_RAT	G3V8W9	Tstd3	Similar to CG12279-PA	1.52	0.000938
D4AB01_RAT	D4AB01	Hint2	Histidine triad nucleotide binding protein 2 (Predicted), isoform CRA_a	1.52	0.002216
CH60_RAT	P63039	Hspd1	60 kDa heat shock protein, mitochondrial	1.52	0.004245
D3ZUI9_RAT	D3ZUI9	Ndufaf8	NADH:ubiquinone oxidoreductase complex assembly factor 8	1.52	0.002759
D3ZT90_RAT	D3ZT90	Gcdh	Glutaryl-CoA dehydrogenase	1.52	0.035278
TM10C_RAT	Q5U2R4	Trmt10c	tRNA methyltransferase 10 homolog C	1.52	0.004136
A0A0G2JW34_RAT	A0A0G2JW34	Cisd3	CDGSH iron sulfur domain 3	1.52	0.013893
D3ZZR9_RAT	D3ZZR9	Fkbp2	Peptidylprolyl isomerase	1.51	0.006559
ACSF2_RAT	Q499N5	Acsf2	Acyl-CoA synthetase family member 2, mitochondrial	1.51	0.004325
D3ZJY1_RAT	D3ZJY1	Mrpl28	Mitochondrial ribosomal protein L28	1.51	0.012429
PREY_RAT	Q5U1Z8	Pyurf	Protein preY, mitochondrial	1.51	0.02374
M0RAK2_RAT	M0RAK2	LOC684270	RCG22622	1.51	0.000417
RM10_RAT	P0C2C4	Mrpl10	39S ribosomal protein L10, mitochondrial	1.51	0.002504
Q3MHT2_RAT	Q3MHT2	Nfs1	Cysteine desulfurase, mitochondrial	1.51	0.0099
SYDM_RAT	Q3KRD0	Dars2	Aspartate—tRNA ligase, mitochondrial	1.51	0.009136
COQ6_RAT	Q68FU7	Coq6	Ubiquinone biosynthesis monooxygenase COQ6, mitochondrial	1.51	0.003601
COX5B_RAT	P12075	Cox5b	Cytochrome c oxidase subunit 5B, mitochondrial	1.51	0.034268
G3V828_RAT	G3V828	Cnpy3	Canopy FGF-signaling regulator 3	1.50	0.011124
M0R7R2_RAT	M0R7R2	LOC683897	Similar to Protein C6orf203	1.50	0.008352
FRDA_RAT	D3ZYW7	Fxn	Frataxin, mitochondrial	1.50	0.005993
A0A0G2JTL5_RAT	A0A0G2JTL5	Pc	Pyruvate carboxylase, mitochondrial	1.50	0.007181
F1LM33_RAT	F1LM33	Lrpprc	Leucine-rich PPR motif-containing protein, mitochondrial	1.50	0.002213
A0A0A0MXZ0_RAT	A0A0A0MXZ0	Isca1	Iron-sulfur cluster assembly 1 homolog, mitochondrial	1.50	0.026833
IDHG1_RAT	P41565	Idh3g	Isocitrate dehydrogenase [NAD] subunit gamma 1, mitochondrial	1.50	0.002832
SYGP1_RAT	Q9QUH6	Syngap1	Ras/Rap GTPase-activating protein SynGAP	0.67	0.004016
A0A0G2JZB8_RAT	A0A0G2JZB8	Gpm6b	Neuronal membrane glycoprotein M6-b	0.66	0.006135
TBB2B_RAT	Q3KRE8	Tubb2b	Tubulin beta-2B chain	0.66	0.010335
D4A1J3_RAT	D4A1J3	Palm3	Paralemmin 3	0.66	0.012666
LDHA_RAT	P04642	Ldha	L-lactate dehydrogenase A chain	0.66	0.001603
A0A096MJW6_RAT	A0A096MJW6	Il1rapl1	Interleukin-1 receptor accessory protein-like 1	0.66	0.021052
A0A0G2K0M8_RAT	A0A0G2K0M8	Ncam1	Neural cell adhesion molecule 1	0.66	0.00849
CLCB_RAT	P08082	Cltb	Clathrin light chain B	0.66	0.005589
F1LR33_RAT	F1LR33	Plppr2	Phospholipid phosphatase-related protein type 2	0.66	0.011704
PALM_RAT	Q920Q0	Palm	Paralemmin-1	0.66	0.007084
F1MA89_RAT	F1MA89	Ccny	Cyclin Y	0.65	0.005391
SV2B_RAT	Q63564	Sv2b	Synaptic vesicle glycoprotein 2B	0.65	0.009948
SC6A1_RAT	P23978	Slc6a1	Sodium- and chloride-dependent GABA transporter 1	0.65	0.002724
FGF14_RAT	Q8R5L7	Fgf14	Fibroblast growth factor 14	0.65	0.017128
Q4V7D9_RAT	Q4V7D9	Smpdl3b	Acid sphingomyelinase-like phosphodiesterase	0.65	0.009225
G3V7A9_RAT	G3V7A9	Cldn10	Claudin	0.65	0.011512
SCN2B_RAT	P54900	Scn2b	Sodium channel subunit beta-2	0.65	0.001306
D4AA77_RAT	D4AA77	Plxnd1	Plexin D1	0.64	0.018219
A0A0G2JVB0_RAT	A0A0G2JVB0	Slc2a3	Solute carrier family 2, facilitated glucose transporter member 3-like	0.63	0.005762
HBA_RAT	P01946	Hba1	Hemoglobin subunit alpha-1/2	0.63	0.001886
ENOB_RAT	P15429	Eno3	Beta-enolase	0.63	0.025177
GRID1_RAT	Q62640	Grid1	Glutamate receptor ionotropic, delta-1	0.63	0.002788
F1M7N2_RAT	F1M7N2	Entpd2	Ectonucleoside triphosphate diphosphohydrolase 2	0.63	0.00134
ALBU_RAT	P02770	Alb	Serum albumin	0.62	0.001197
Q499T3_RAT	Q499T3	Sirpa	Sirpa protein	0.62	0.008158
2ABG_RAT	P97888	Ppp2r2c	Serine/threonine-protein phosphatase 2A 55 kDa regulatory subunit B gamma isoform	0.62	0.027676
A0A0G2JTH4_RAT	A0A0G2JTH4	Cd47	Leukocyte surface antigen CD47	0.61	0.011316
M0RBJ0_RAT	M0RBJ0	Gng2	Guanine nucleotide-binding protein subunit gamma	0.61	0.003203
G3V6R0_RAT	G3V6R0	Slc1a2	Amino acid transporter	0.61	0.008327
CRYM_RAT	Q9QYU4	Crym	Ketimine reductase mu-crystallin	0.61	0.010508
NRN1_RAT	O08957	Nrn1	Neuritin	0.60	0.003871
GPM6A_RAT	Q812E9	Gpm6a	Neuronal membrane glycoprotein M6-a	0.58	0.005952
KCIP2_RAT	Q9JM59	Kcnip2	Kv channel-interacting protein 2	0.55	0.013844
F1M9G9_RAT	F1M9G9	Scn2a	Sodium channel protein	0.51	0.008815
E9PSV8_RAT	E9PSV8	Gpm6b	Neuronal membrane glycoprotein M6-b	0.45	0.034939

### Bioinformatics analysis of DEPs in mitochondrial proteome

In order to have a functional overview of the DEPs, we performed function annotation by GO and KEGG analyses. The most relevant and significant enriched terms and pathways (FDR<0.01) were illustrated by biological process (BP) (Table 2), cellular compartment (CC) (Table 3), molecular function (MF) (Table 4), and KEGG pathway (Table 5) separately. The GO and KEGG Term ID, Term, Rich factors (Ratio), Enrichment, FDR and Protein IDs included were all listed in these tables. The most significantly enriched GO terms in the biological process category were mainly annotated with the terms tricarboxylic acid (TCA) cycle (GO: 0006099, 13 proteins), fatty acid beta-oxidation using acyl-CoA dehydrogenase (GO: 0033539, 7 proteins), 2-oxoglutarate metabolic process (GO: 0006103, 5 proteins), etc. DEPs classified in the cellular component category were mainly annotated with the terms mitochondrion (GO: 0005739, 75 proteins) and mitochondrial matrix (GO: 0005759, 28 proteins). DEPs classified in the molecular function category were mainly annotated with the terms flavin adenine dinucleotide binding (GO: 0050660, 8 proteins), pyridoxal phosphate binding (GO: 0030170, 8 proteins), NAD binding (GO: 0051287, 8 proteins) and Succinate-CoA ligase (ADP-forming) activity (GO: 0004775, 3 proteins). The 163 DEPs were annotated with KEGG pathways, and the top 3 most enriched pathways were carbon metabolism (path: rno01200, 19 proteins), valine, leucine and isoleucine degradation (path: rno00280, 12 proteins) and metabolic pathways (path: rno01100, 40 proteins), and the citrate cycle (TCA cycle) was one of the significantly enriched metabolic pathways.

**Table 2:** The significantly enriched GO terms related to biological processes.

Term ID	Term	Ratio	Enrichment	FDR	Protein IDs
GO:0006099	tricarboxylic acid cycle	0.52	12.10	4.40E-10	ACON_RAT,MDHM_RAT,F1LM47_RAT,F1LNF7_RAT,IDH3B_RAT,FUMH_RAT,IDHG1_RAT,G3V6P2_RAT,IDHP_RAT,G3V936_RAT,A0A0H2UHE1_RAT,F1LPV8_RAT,DHTK1_RAT
GO:0033539	fatty acid beta-oxidation using acyl-CoA dehydrogenase	0.78	8.44	1.01E-06	ETFA_RAT,IVD_RAT,ACADL_RAT,ETFB_RAT,D3ZT90_RAT,G3V796_RAT,Q6IMX3_RAT
GO:0006103	2-oxoglutarate metabolic process	0.63	5.40	0.000742	AATM_RAT,DLDH_RAT,IDH3B_RAT,IDHG1_RAT,IDHP_RAT
GO:0006102	isocitrate metabolic process	0.80	4.99	0.001418	ACON_RAT,IDH3B_RAT,IDHG1_RAT,IDHP_RAT
GO:0019254	carnitine metabolic process, CoA-linked	1.00	4.25	0.006217	ACADL_RAT,A0A0H2UI21_RAT,G3V796_RAT

**Table 3:** The significantly enriched GO terms related to cellular component.

Term ID	Term	Ratio	Enrichment	FDR	Protein IDs	Accession
GO:0005739	mitochondrion	0.16	30.24	7.92E-29	ACON_RAT,CH60_RAT,DHE3_RAT,A0A0G2JTL5_RAT,GABT_RAT,MDHM_RAT,AATM_RAT,THIL_RAT,DLDH_RAT,F1LM47_RAT,F1LNF7_RAT,G3V945_RAT,FUMH_RAT,IDHG1_RAT,SCOT1_RAT,G3V7J0_RAT,A0A0G2JSS8_RAT,G3V6P2_RAT,F1M5N4_RAT,IDHP_RAT,A0A0H2UHE1_RAT,G3V7I5_RAT,ETFA_RAT,AL7A1_RAT,IVD_RAT,ACSF2_RAT,ES1_RAT,3HIDH_RAT,AUHM_RAT,ACADL_RAT,G3V9U2_RAT,OAT_RAT,A0A0H2UI21_RAT,ECHM_RAT,F1LPV8_RAT,C1QBP_RAT,SODM_RAT,CH10_RAT,D4AB01_RAT,Q5U3Z7_RAT,ATIF1_RAT,MAAI_RAT,G3V7I0_RAT,ECH1_RAT,TRXR2_RAT,A0A0G2JUZ5_RAT,CATB_RAT,PPIF_RAT,DHTK1_RAT,D4ADD7_RAT,COQ6_RAT,FAHD1_RAT,GCSH_RAT,GATA_RAT,D3ZT90_RAT,G3V796_RAT,TM10C_RAT,HMCS2_RAT,D4A833_RAT,Q6IMX3_RAT,Q6AY99_RAT,D3ZUI9_RAT,F1M8H2_RAT,D3ZT98_RAT,PREY_RAT,FMT_RAT,FRDA_RAT,HOT_RAT,A0A0G2K2Q2_RAT,A0A0G2K7D7_RAT,M0R4L6_RAT,A0A0G2K9G3_RAT,F210A_RAT,G3V8U8_RAT,SDHF1_RAT	Q9ER34,P63039,P10860,A0A0G2JTL5,P50554,P04636,P00507,P17764,Q6P6R2,F1LM47,F1LNF7,G3V945,P14408,P41565,B2GV06,G3V7J0,A0A0G2JSS8,G3V6P2,F1M5N4,P56574,A0A0H2UHE1,G3V7I5,P13803,Q64057,P12007,Q499N5,P56571,P29266,F1LU71,P15650,G3V9U2,P04182,A0A0H2UI21,P14604,F1LPV8,O35796,P07895,P26772,D4AB01,Q5U3Z7,Q03344,P57113,G3V7I0,Q62651,Q9Z0J5,A0A0G2JUZ5,P00787,P29117,Q4KLP0,D4ADD7,Q68FU7,Q6AYQ8,Q5I0P2,Q5FWT5,D3ZT90,G3V796,Q5U2R4,P22791,D4A833,Q6IMX3,Q6AY99,D3ZUI9,F1M8H2,D3ZT98,Q5U1Z8,Q5I0C5,D3ZYW7,Q4QQW3,A0A0G2K2Q2,A0A0G2K7D7,M0R4L6,A0A0G2K9G3,Q5XIJ4,G3V8U8,B0K036
GO:0005759	mitochondrial matrix	0.33	18.83	1.02E-17	CH60_RAT,DHE3_RAT,GABT_RAT,MDHM_RAT,AATM_RAT,THIL_RAT,DLDH_RAT,SCOT1_RAT,G3V936_RAT,ETFA_RAT,IVD_RAT,ACADL_RAT,OAT_RAT,ETFB_RAT,ECHM_RAT,C1QBP_RAT,CH10_RAT,THTR_RAT,Q5U3Z7_RAT,F1LP30_RAT,A0A0G2JZA2_RAT,PPIF_RAT,D4ADD7_RAT,HMCS2_RAT,SYDM_RAT,F1M8H2_RAT,D4A7X5_RAT,SDHF1_RAT	P63039,P10860,P50554,P04636,P00507,P17764,Q6P6R2,B2GV06,G3V936,P13803,P12007,P15650,P04182,Q68FU3,P14604,O35796,P26772,P24329,Q5U3Z7,F1LP30,A0A0G2JZA2,P29117,D4ADD7,P22791,Q3KRD0,F1M8H2,D4A7X5,B0K036

**Table 4:** The significantly enriched GO terms related to molecular function.

Term ID	Term	Ratio	Enrichment	FDR	Protein IDs
GO:0050660	flavin adenine dinucleotide binding	0.35	5.70	0.000539659	DLDH_RAT,ETFA_RAT,IVD_RAT,ACADL_RAT,TRXR2_RAT,D3ZT90_RAT,G3V796_RAT,Q6IMX3_RAT
GO:0030170	pyridoxal phosphate binding	0.30	5.11	0.00106165	GABT_RAT,AATM_RAT,ALBU_RAT,OAT_RAT,Q5U3Z7_RAT,Q3MHT2_RAT,A0A0G2JUZ5_RAT,A0A0G2K2Q2_RAT
GO:0051287	NAD binding	0.26	4.62	0.002185681	DLDH_RAT,F1LNF7_RAT,IDH3B_RAT,IDHG1_RAT,F1M5N4_RAT,IDHP_RAT,3HIDH_RAT,LDHA_RAT
GO:0004775	succinate-CoA ligase (ADP-forming) activity	1.00	4.16	0.004661018	F1LM47_RAT,A0A0H2UHE1_RAT,F1LPV8_RAT

**Table 5:** The significantly enriched KEGG pathway from the DEPs.

Term ID	Term	Ratio	Enrichment	FDR	Protein IDs
path:rno01200	Carbon metabolism	0.33	12.58	2.92E-11	ACON_RAT,DHE3_RAT,MDHM_RAT,AATM_RAT,THIL_RAT,DLDH_RAT,F1LM47_RAT,IDH3B_RAT,G3V7J0_RAT,G3V6P2_RAT,F1M5N4_RAT,IDHP_RAT,Q68FZ8_RAT,ECHM_RAT,ENOB_RAT,Q5U3Z7_RAT,GCSH_RAT,G3V796_RAT,Q6IMX3_RAT
path:rno00280	Valine, leucine and isoleucine degradation	0.46	9.86	7.66E-09	GABT_RAT,THIL_RAT,DLDH_RAT,SCOT1_RAT,G3V7J0_RAT,Q68FZ8_RAT,AL7A1_RAT,IVD_RAT,3HIDH_RAT,ECHM_RAT,G3V796_RAT,Q6IMX3_RAT
path:rno01100	Metabolic pathways	0.11	9.49	1.19E-08	ACON_RAT,DHE3_RAT,GABT_RAT,MDHM_RAT,AATM_RAT,THIL_RAT,DLDH_RAT,F1LM47_RAT,G3V945_RAT,IDH3B_RAT,G3V7J0_RAT,G3V6P2_RAT,F1M5N4_RAT,IDHP_RAT,Q68FZ8_RAT,AL7A1_RAT,IVD_RAT,3HIDH_RAT,ACADL_RAT,OAT_RAT,LDHA_RAT,ECHM_RAT,COX5B_RAT,ENOB_RAT,THTR_RAT,Q5U3Z7_RAT,Q3MHT2_RAT,MAAI_RAT,DHTK1_RAT,COQ6_RAT,FAHD1_RAT,GCSH_RAT,GATA_RAT,D3ZT90_RAT,G3V796_RAT,Q6IMX3_RAT,Q6AY99_RAT,M0R4L6_RAT,CEGT_RAT,ATP6_RAT
path:rno00640	Propanoate metabolism	0.50	7.83	4.13E-07	GABT_RAT,THIL_RAT,DLDH_RAT,F1LM47_RAT,G3V7J0_RAT,Q68FZ8_RAT,LDHA_RAT,ECHM_RAT,G3V796_RAT
path:rno00310	Lysine degradation	0.47	5.89	2.86E-05	THIL_RAT,DLDH_RAT,G3V6P2_RAT,AL7A1_RAT,ECHM_RAT,DHTK1_RAT,D3ZT90_RAT
path:rno00380	Tryptophan metabolism	0.44	5.66	3.51E-05	THIL_RAT,DLDH_RAT,G3V6P2_RAT,AL7A1_RAT,ECHM_RAT,DHTK1_RAT,D3ZT90_RAT
path:rno00630	Glyoxylate and dicarboxylate metabolism	0.44	5.66	3.51E-05	ACON_RAT,MDHM_RAT,THIL_RAT,DLDH_RAT,Q68FZ8_RAT,Q5U3Z7_RAT,GCSH_RAT
path:rno00020	Citrate cycle (TCA cycle)	0.41	5.44	5.03E-05	ACON_RAT,MDHM_RAT,DLDH_RAT,F1LM47_RAT,IDH3B_RAT,G3V6P2_RAT,IDHP_RAT
path:rno00650	Butanoate metabolism	0.46	5.06	0.000107806	GABT_RAT,THIL_RAT,G3V945_RAT,SCOT1_RAT,ECHM_RAT,Q6IMX3_RAT
path:rno00071	Fatty acid degradation	0.32	4.57	0.000295395	THIL_RAT,AL7A1_RAT,ACADL_RAT,ECHM_RAT,D3ZT90_RAT,G3V796_RAT,Q6IMX3_RAT
path:rno00620	Pyruvate metabolism	0.35	4.26	0.000551788	MDHM_RAT,THIL_RAT,DLDH_RAT,F1M5N4_RAT,AL7A1_RAT,LDHA_RAT
path:rno00410	beta-Alanine metabolism	0.36	3.64	0.002124661	GABT_RAT,G3V7J0_RAT,AL7A1_RAT,ECHM_RAT,G3V796_RAT
path:rno01210	2-Oxocarboxylic acid metabolism	0.40	3.20	0.005443792	ACON_RAT,AATM_RAT,IDH3B_RAT,IDHP_RAT
path:rno01200	Carbon metabolism	0.33	12.58	2.92E-11	ACON_RAT,DHE3_RAT,MDHM_RAT,AATM_RAT,THIL_RAT,DLDH_RAT,F1LM47_RAT,IDH3B_RAT,G3V7J0_RAT,G3V6P2_RAT,F1M5N4_RAT,IDHP_RAT,Q68FZ8_RAT,ECHM_RAT,ENOB_RAT,Q5U3Z7_RAT,GCSH_RAT,G3V796_RAT,Q6IMX3_RAT

**Fig 3:**
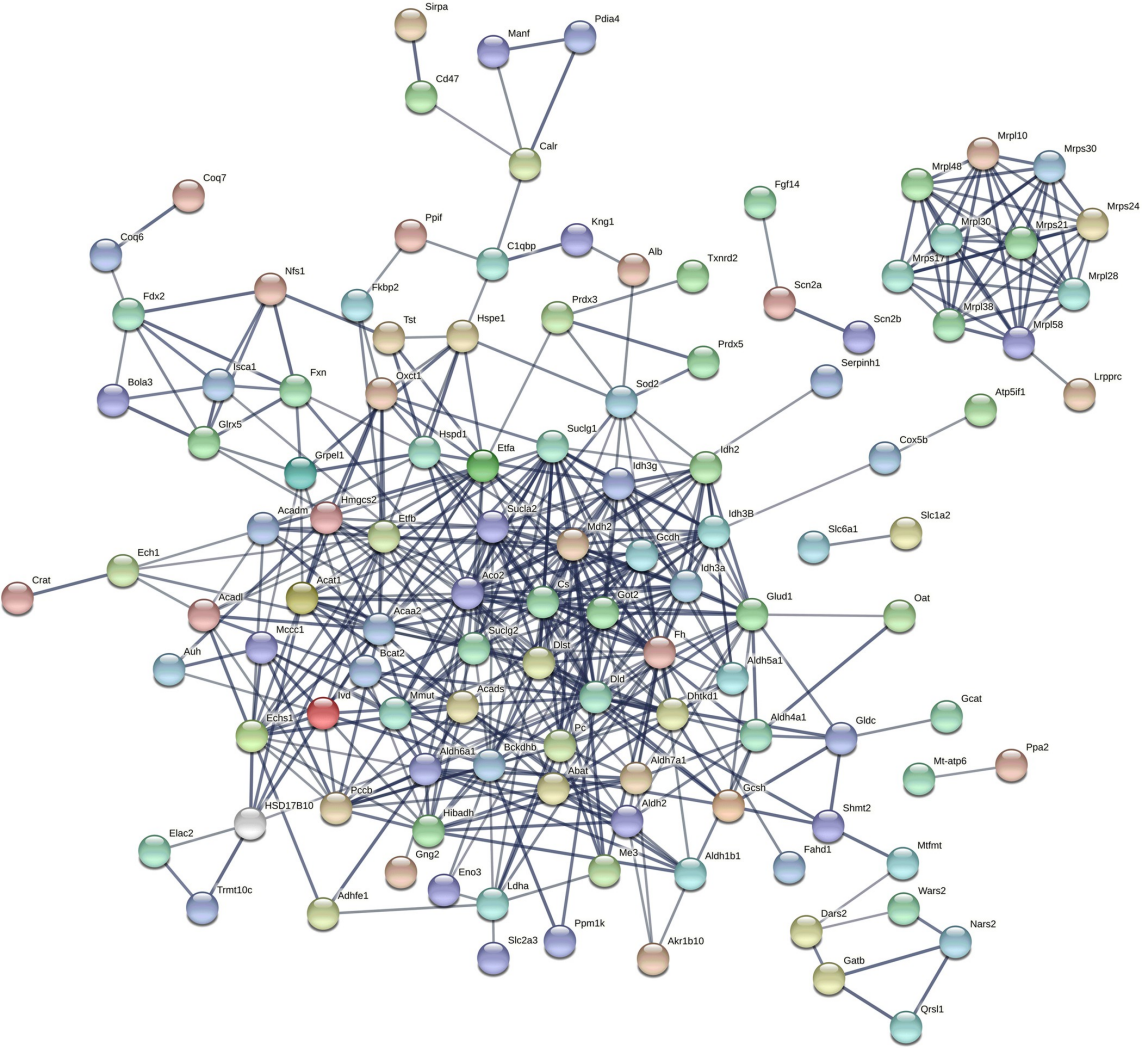
The protein-protein interaction (PPI) network. The colored nodes represented query proteins and first shell of interactors, whereas, white nodes represented second shell of interactors. Lines represented the interactions between two nodes.

Next, we constructed the protein-protein interaction (PPI) network to screen for hub proteins (**Fig 3**). The top 10 high-degree hub nodes included DLD (Dihydrolipoyl dehydrogenase), CS (Citrate synthase), ACO2 (Aconitate hydratase), MDH2 (Malate dehydrogenase), DLST (Dihydrolipoamide S-succinyltransferase), IDH3A (Isocitrate dehydrogenase [NAD] subunit), ALDH6A1 (Aldehyde dehydrogenase family 6, subfamily A1, isoform CRA_b), FH (Fumarate hydratase), GLUD1 (Glutamate dehydrogenase 1), and GOT2 (Aspartate aminotransferase), and they may play an important role in mediating the effects of SM on the mitochondrial metabolic function. These data indicate that the effects of SM on mitochondria are mainly in the pathway of material metabolism and energy metabolism.

### Validation of the selected proteins in TCA by Western blotting

**Fig 4:**
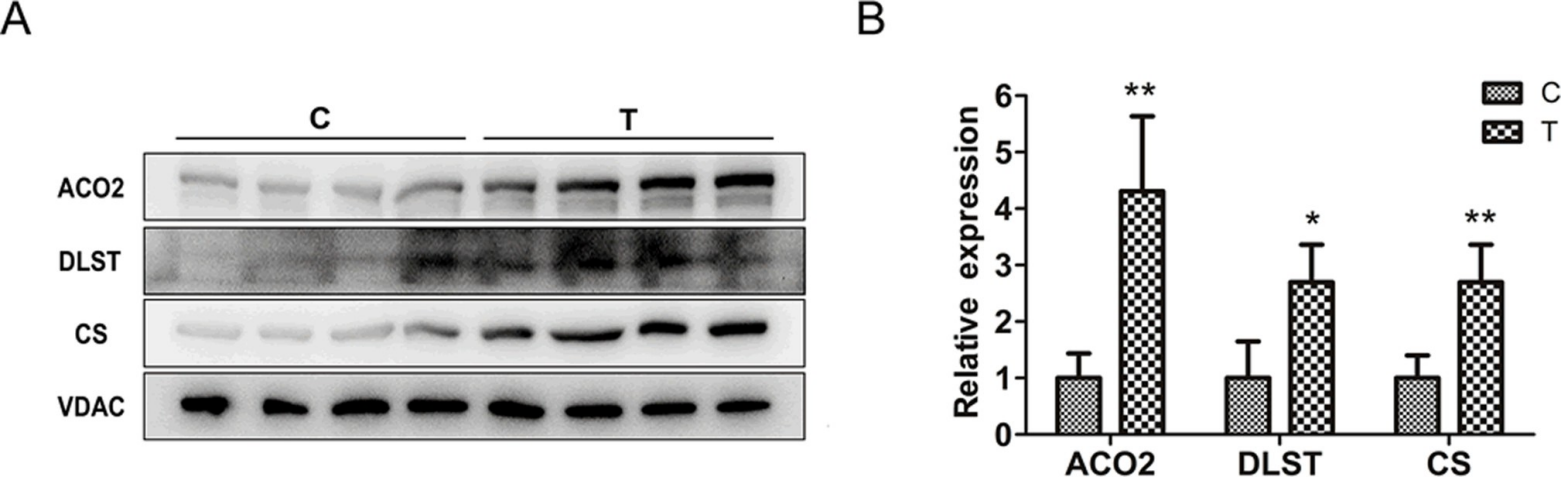
28-day tail suspension promotes the expression of ACO2, DLST and CS in hippocampus mitochondria of rats. **(A)** Protein levels of ACO2, DLST and CS were determined by immunoblotting. **(B)** The relative expressions of ACO2, DLST and CS were represented by the intensity ratio between the protein and the loading control (VDAC) in each lane. n = 4, error bars indicated standard deviations. *p<0.05; **p<0.01 compared with each control group.

As the tricarboxylic acid (TCA) cycle was the most enriched GO terms in the biological process category, we used Western blotting to verify the relative expressions of the 3 proteins, ACO2 DLST and CS. All of the 3 proteins were involved in TCA cycle and were the hub genes in PPI network we constructed. The mass spectrometry results showed that their mean expression levels reached 1.71 (ACO2), 1.53 (DLST), and 1.85 (CS) times that of the Control group (**Table 1**). The Western blotting results showed that after 28 days of tail suspension, all three proteins were significantly upregulated, and the relative expressions of ACO2, DLST and CS reached 4.31, 2.70 and 2.70 times that of the corresponding control group, respectively (**Fig 4**). This suggests that SM greatly promotes the function of TCA cycle.

## Discussion

In this study, we found that 28 days of tail suspension increased the number and size of mitochondria in the hippocampus of rats and TMT-based proteomics analysis revealed 128 mitochondrial proteins upregulation and 35 mitochondrial proteins downregulation. Bioinformatics analysis implied that mitochondrial metabolic pathways related TCA cycle and fatty acid oxidation were significantly changed. We verified the upregulated expressions of three TCA cycle related proteins, ACO2, DLST and CS. Our study suggests that SM can cause mitochondrial dynamic and metabolic function related proteins changes, which may be one of the mechanisms of the effects of space microgravity on brain function.

Previously, Mikheeva et al. reported that after 30 days flight on the Bion-M1 biosatellite, the number and size of mitochondria in the soma of motoneurons and in axons coming from the vestibular structures increased in mouse [22]. Tan et al. showed that when compared with cells maintained under normal gravity, BL6-10 cells treated with simulating microgravity showed higher mitochondrial content and more abundant cytoplasmic mitochondria, and significantly reduced glycolytic metabolism [23]. In consistent with these findings, our study showed that the mitochondrial number and size of rat hippocampus were increased after 28 days of tail suspension. As a highly dynamic organelle, the function of mitochondria is dynamically regulated by the fission and fusion in various cell types, thus regulating the morphology, quantity, distribution, metabolism and biogenesis of mitochondria [17]. Some studies indicated that mitochondrial division can enhance its function. Fulghum and Hill found that catecholamines promotes mitochondrial fission and up-regulates PGC1α, thereby dramatically increasing mitochondrial function and long-term increase in mitochondrial abundance and fatty acid oxidation capacity [24]. Rana et al. found that promoting mitochondrial fission in midlife Drosophila can improve multiple markers of mitochondrial function and reduce mitochondrial ROS levels [25]. In current study, the analysis of DEPs revealed that most of the proteins (128/163) were upregulated after tail suspension, suggesting that SM may enhance the function of mitochondria in the hippocampus possibly through influencing the fission of mitochondria, and this mechanism requires further investigation.

The GO analysis of our current study showed that the most significantly enriched category in the cellular component were TCA cycle, fatty acid beta-oxidation using acyl-CoA dehydrogenase, 2-oxoglutarate metabolic process, isocitrate metabolic process and carnitine metabolic process, CoA-linked, and all of them are the important processes of mitochondria. These findings suggested that the DEPs are mainly involved in mitochondria and energy metabolism. In the TCA cycle pathway, all 13 identified proteins were upregulated. Similarly, the DEPs that involved in fatty acid beta-oxidation using acyl-CoA dehydrogenase process were also upregulated. Espinosa-Jeffrey et al. used 3D-clinostat robot to simulate the microgravity in oligodendrocytes, and found that the mitochondrial respiration and glycolysis are increased after 24 hours exposure to SM, indicating that SM enhances the mitochondrial function [26]. In another study, the primary osteoblasts were exposed to SM for 110 hours, and the metabonomics and proteomics results showed that TCA cycle is activated and acetyl coenzyme A is accumulated [27]. The real flight data also showed that microgravity increases the TCA activity. da Silveira et al. analyzed four human cell lines (fibroblasts, endothelial cells, primary T cells, and hair follicles) in vitro datasets available on GeneLab by gene set enrichment analysis (GSEA) for the overlapping pathways, and found one overlapping collection of gene sets across all four cell types, which contains four mitochondrial function gene ontology (GO) terms: mitochondrial ATP synthesis, mitochondrial electron transport, oxidative phosphorylation (OXPHOS), and hydrogen ion transmembrane transportation. They next analyzed the metabonomics data of gastrocnemius and quadriceps femoris muscles in mice after 35 days spaceflight, and the enrichment analysis showed that spaceflight increases the mitochondrial and energy metabolism related pathways, such as the b-oxidation of long-chain fatty acids and the TCA cycle [14]. The famous NASA twins study demonstrated that the levels of plasma TCA cycle intermediates (citric acid and malic acid) are raised during flight compared with pre-flight and post-flight levels [28], indicating that the TCA activity is elevated. Our results suggested that mitochondrial activity and energy metabolism are remarkably upregulated in rat hippocampus after 28-day tail suspension. Although the organizations studied are not the same, their results confirmed that our findings are similar to those in the real space microgravity environment. Material metabolism and energy metabolism are the main functions of mitochondria. The KEGG pathway analysis of our results indicated that 14 metabolic pathways showed significant differences between Control group and SM group. Almost all the material and energy metabolism, including carbon metabolism, amino acid metabolism (e.g., valine, leucine and isoleucine degradation, lysine degradation, tryptophan metabolism and beta-Alanine metabolism), TCA cycle and lipid metabolism (e.g., fatty acid degradation), have been changed significantly in our study, further proving that tail suspension can change the function of hippocampal mitochondria in rats.

Protein-protein interaction (PPI) research can reveal the protein function of DEPs at the molecular level, and explain the cellular mechanism by elucidating the interaction of whole genome proteins [29]. In this study, we constructed the PPI network of DEPs, and found that 6 (CS, ACO2, MDH2, DLST, IDH3A and FH) of top 10 high-degree hub nodes were involved in TCA cycle. In particular, we used Western blotting to detect the expressions of ACO2, CS and DLST, and found that their expression trends were consistent with the results of proteomics. Considering that ACO2 belongs to aconitase family and plays an important role in maintaining oxidative phosphorylation and energy generation [30, 31], we believe that other nodal genes are also involved in the regulation of mitochondrial function or the downstream gene expression or metabolism by microgravity. Our proteomics analysis also showed that the expressions of antioxidant enzymes such as SOD2 and prdx3 were increased, suggesting that SM may induce the transfer of energy metabolism from glycolysis to oxidative phosphorylation in rat hippocampus. This may reflect the compensation of body to the harmful effects of SM.

The future success of long-term space exploration requires a comprehensive understanding of the impact of spaceflight on human biology. After analyzing the samples from 59 astronauts and hundreds of samples flown in space by transcriptomics, proteomics, metabolomics and epigenetics, da Silveira et al. concluded that mitochondrial disorders are the central hub of space biology [14]. In view of the fact that true microgravity cannot be simulated on the earth, the tail suspension model only simulate the fluid shift, muscle atrophy, bone loss effects in microgravity. In our study, the effects of SM on the dynamics and proteomics of mouse hippocampal mitochondria may be the result of long-term body fluid shift. In order to ensure the health of human spaceflight, more in-depth experimental research on mitochondrial functions and molecular mechanisms are needed in the aerospace environment.

## Supporting information

S1 Fig Uncropped images of Western blots in Fig 4. (TIF)

S1 Table Raw data of proteomics. (XLSX)
