# Supplementary figures and images for: The mitochondrial proteomic changes of rat hippocampus induced by 28-day simulated microgravity
Source: PLoS One. 2022 Mar 10;17(3):e0265108. doi: 10.1371/journal.pone.0265108 (PMC8912132; doi:10.1371/journal.pone.0265108)

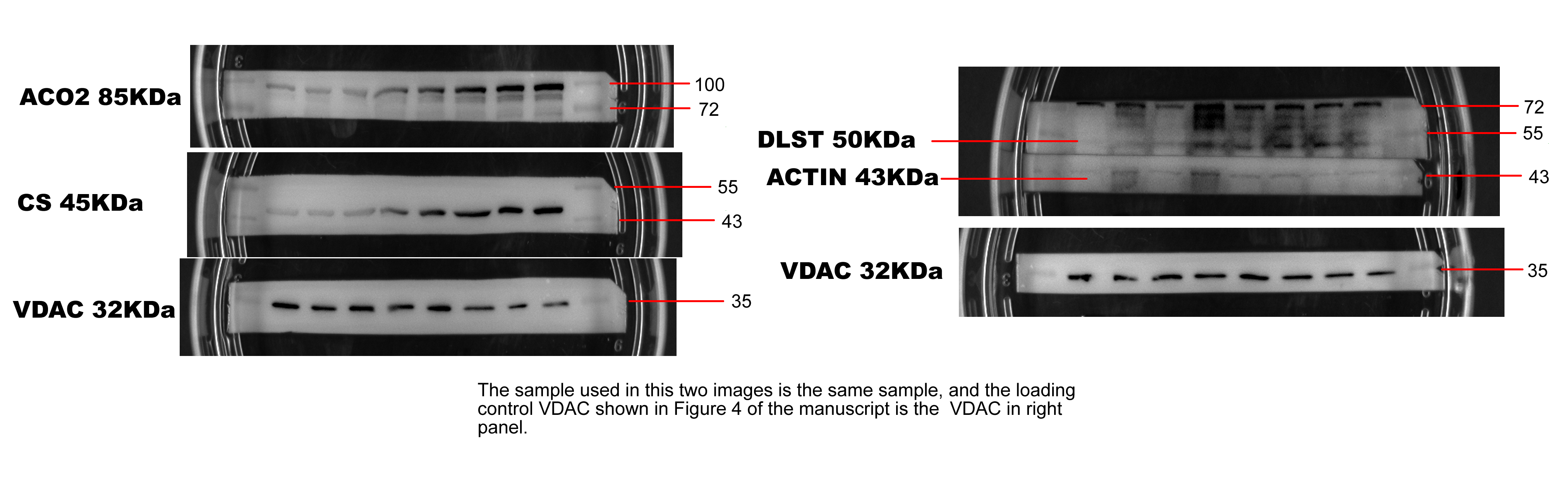

Supplement: S1 Fig — (TIF) [file pone.0265108.s001.tif]
