# Supplementary material for: The mitochondrial proteomic changes of rat hippocampus induced by 28-day simulated microgravity
Source: PLoS One. 2022 Mar 10;17(3):e0265108. doi: 10.1371/journal.pone.0265108 (PMC8912132; doi:10.1371/journal.pone.0265108)
Supplement: Attachment — Submitted filename: Response to Reviewers.docx [file pone.0265108.s003.docx]

Date:	Dec 01 2021 01:43PM

To:	"Guohua Ji" jgh1682004@126.com

cc:	angela.chambery@unicampania.it

From:	"PLOS ONE" plosone@plos.org

Subject:	PLOS ONE Decision: Revision required [PONE-D-21-31461]

PONE-D-21-31461

The mitochondrial proteomic changes of rat hippocampus induced by 28-day simulated microgravity

PLOS ONE

Dear Dr. Ji,

Thank you for submitting your manuscript to PLOS ONE. After careful consideration, we feel that it has merit but does not fully meet PLOS ONE’s publication criteria as it currently stands. Therefore, we invite you to submit a revised version of the manuscript that addresses the points raised during the review process.

Both reviewers appreciate rigor and results of your paper. However, some weaknesses and concerns were expressed by both referees mainly regarding the focus of the study and discussion of results. Since no functional analyses neither data on the morphology of mitochondria are provided in support of proteomic data, authors are encouraged to correct the text or adequate the experimental design to support conclusions. Some experimental details should be better described in the Methods section.

Please submit your revised manuscript by Jan 15 2022 11:59PM. If you will need more time than this to complete your revisions, please reply to this message or contact the journal office at plosone@plos.org. When you're ready to submit your revision, log on to https://www.editorialmanager.com/pone/ and select the 'Submissions Needing Revision' folder to locate your manuscript file.

Please include the following items when submitting your revised manuscript:

A rebuttal letter that responds to each point raised by the academic editor and reviewer(s). You should upload this letter as a separate file labeled 'Response to Reviewers'.

A marked-up copy of your manuscript that highlights changes made to the original version. You should upload this as a separate file labeled 'Revised Manuscript with Track Changes'.

An unmarked version of your revised paper without tracked changes. You should upload this as a separate file labeled 'Manuscript'.

If you would like to make changes to your financial disclosure, please include your updated statement in your cover letter. Guidelines for resubmitting your figure files are available below the reviewer comments at the end of this letter.

If applicable, we recommend that you deposit your laboratory protocols in protocols.io to enhance the reproducibility of your results. Protocols.io assigns your protocol its own identifier (DOI) so that it can be cited independently in the future. For instructions see: https://journals.plos.org/plosone/s/submission-guidelines#loc-laboratory-protocols. Additionally, PLOS ONE offers an option for publishing peer-reviewed Lab Protocol articles, which describe protocols hosted on protocols.io. Read more information on sharing protocols at https://plos.org/protocols?utm_medium=editorial-email&utm_source=authorletters&utm_campaign=protocols.

We look forward to receiving your revised manuscript.

Kind regards,

Angela Chambery, PhD

Academic Editor

PLOS ONE

Journal Requirements:

When submitting your revision, we need you to address these additional requirements.

1. Please ensure that your manuscript meets PLOS ONE's style requirements, including those for file naming. The PLOS ONE style templates can be found at

https://journals.plos.org/plosone/s/file?id=wjVg/PLOSOne_formatting_sample_main_body.pdf and

<https://journals.plos.org/plosone/s/file?id=ba62/PLOSOne_formatting_sample_title_authors_affiliations.pdf>.

**Reply: We confirmed that our manuscript meets PLOS ONE's style requirements.**

2. In your Methods section, please provide additional information on the animal research and ensure you have included details on : (1) methods of sacrifice (2) methods of anesthesia and/or analgesia, and (2) efforts to alleviate suffering.

**Reply: Information on (1) methods of sacrifice, (2) methods of anesthesia and/or analgesia, (3) efforts to alleviate suffering were added to the “Animals and SM model construction” and “Transmission electron microscopy” sections in the Material and Methods section.**

3. Thank you for stating the following in the Acknowledgments Section of your manuscript:

[This work was supported by The Foundation of State Key Laboratory of Space Medicine Fundamentals and Application, China Astronaut Research and Training Center (SMFA17A03, SMFA19B02, SMFA19K08, SMFA17B09), The National Natural Science Foundation of China (81773930, 21635001, 31800707, 31800998). The funders had no role in the study design, data collection and analysis, decision to publish, and preparation of the manuscript.]

We note that you have provided funding information that is currently declared in your Funding Statement. However, funding information should not appear in the Acknowledgments section or other areas of your manuscript. We will only publish funding information present in the Funding Statement section of the online submission form.

Please remove any funding-related text from the manuscript and let us know how you would like to update your Funding Statement. Currently, your Funding Statement reads as follows:

[This work was supported by The Foundation of State Key Laboratory of Space Medicine Fundamentals and Application, China Astronaut Research and Training Center (SMFA17A03, SMFA19B02, SMFA19K08, SMFA17B09), The National Natural Science Foundation of China (81773930, 21635001, 31800707, 31800998). The funders had no role in the study design, data collection and analysis, decision to publish, and preparation of the manuscript. The funders had no role in study design, data collection and analysis, decision to publish, or preparation of the manuscript.]

Please include your amended statements within your cover letter; we will change the online submission form on your behalf.

**Reply: Thanks for reminding. We have deleted the Acknowledgments section and the funding information in the manuscript and include the amended statements in the cover letter.**

4. We note that you have stated that you will provide repository information for your data at acceptance. Should your manuscript be accepted for publication, we will hold it until you provide the relevant accession numbers or DOIs necessary to access your data. If you wish to make changes to your Data Availability statement, please describe these changes in your cover letter and we will update your Data Availability statement to reflect the information you provide.

**Reply: We have uploaded the original proteomic data as an attachment named S1_Raw_data. So the Data Availability statement can be changed to “All relevant data are within the manuscript and its Supporting Information files”.**

5. PLOS ONE now requires that authors provide the original uncropped and unadjusted images underlying all blot or gel results reported in a submission’s figures or Supporting Information files. This policy and the journal’s other requirements for blot/gel reporting and figure preparation are described in detail at https://journals.plos.org/plosone/s/figures#loc-blot-and-gel-reporting-requirements and https://journals.plos.org/plosone/s/figures#loc-preparing-figures-from-image-files. When you submit your revised manuscript, please ensure that your figures adhere fully to these guidelines and provide the original underlying images for all blot or gel data reported in your submission. See the following link for instructions on providing the original image data: https://journals.plos.org/plosone/s/figures#loc-original-images-for-blots-and-gels.

In your cover letter, please note whether your blot/gel image data are in Supporting Information or posted at a public data repository, provide the repository URL if relevant, and provide specific details as to which raw blot/gel images, if any, are not available. Email us at plosone@plos.org if you have any questions.

**Response: We have provided the original underlying images for Figure 4 in our manuscript with the name of S2_raw_images.**

Additional Editor Comments:

Two expert reviewers have assessed your submission and feel that it has potential for publication, and so I would like to invite you to revise the paper.

Both reviewers appreciate rigor and results of your paper. However, some weaknesses and concerns were expressed by both referees mainly regarding the focus of the study and discussion of results. Since no functional analyses neither data on morphology of mitochondria are provided in support of proteomic data, authors are encouraged to correct the text or adequate the experimental design to support conclusions. Some experimental details should be better described in the Methods section.

**Reply: Thank you. In order to make the article more rigorous, we have revised the discussion section to make it more focused; mitochondrial area and diameter were analyzed to support proteomic data; Part of the text has been corrected to support the conclusion; some experimental details are better described. Please refer to the revised manuscript for details.**

[Note: HTML markup is below. Please do not edit.]

Reviewers' comments:

Reviewer's Responses to Questions

Comments to the Author

1. Is the manuscript technically sound, and do the data support the conclusions?

The manuscript must describe a technically sound piece of scientific research with data that supports the conclusions. Experiments must have been conducted rigorously, with appropriate controls, replication, and sample sizes. The conclusions must be drawn appropriately based on the data presented.

Reviewer #1: Partly

Reviewer #2: Partly

2. Has the statistical analysis been performed appropriately and rigorously?

Reviewer #1: Yes

Reviewer #2: Yes

3. Have the authors made all data underlying the findings in their manuscript fully available?

The PLOS Data policy requires authors to make all data underlying the findings described in their manuscript fully available without restriction, with rare exception (please refer to the Data Availability Statement in the manuscript PDF file). The data should be provided as part of the manuscript or its supporting information, or deposited to a public repository. For example, in addition to summary statistics, the data points behind means, medians and variance measures should be available. If there are restrictions on publicly sharing data—e.g. participant privacy or use of data from a third party—those must be specified.

Reviewer #1: Yes

Reviewer #2: Yes

4. Is the manuscript presented in an intelligible fashion and written in standard English?

PLOS ONE does not copyedit accepted manuscripts, so the language in submitted articles must be clear, correct, and unambiguous. Any typographical or grammatical errors should be corrected at revision, so please note any specific errors here.

Reviewer #1: Yes

Reviewer #2: Yes

5. Review Comments to the Author

Please use the space provided to explain your answers to the questions above. You may also include additional comments for the author, including concerns about dual publication, research ethics, or publication ethics. (Please upload your review as an attachment if it exceeds 20,000 characters)

Reviewer #1: Guohua Ji and coworkers present an original study focusing on the effects of simulated microgravity (by tail suspension for 28 days) on mitochondrial proteome in rat hippocampus.

The search for mitochondrial alterations in cognitive function decline caused by the aerospace microgravity environment represents the originality of the study.

The study was conducted with rigor and the results were well presented, however some weaknesses must be considered:

1) The authors claim to have obtained information on mitochondrial morphology and dynamics. However no morphometric analysis were done. Morphology should be “quantitatively” described by mitochondrial size measurements, reporting data on the mitochondrial mean surface area and Feret's diameter, for example.

**Reply: Thank you for your suggestion. We analyzed the original picture of transmission electron microscope, and attached the results of the average surface area and Feret's diameter of mitochondria in the manuscript.**

2) The authors claim to have clearly observed in the hippocampus of T animals mitochondrial swelling, and cristae loos. This, together with an increase in mitochondrial number, lead the authors to state in favor of mitochondrial fission and altered mitochondrial dynamics. However, no data have been furnished on expression levels or activation of key markers of mitochondrial biogenesis and dynamics, such as PGC1α, mitofusins, OPA1 and DRP1.

So, conclusion on mitochondrial morphology and dynamics are not at all supported by the reported data and remain speculative.

**Reply: Thank you for your comments. We changed the word "fission" in the abstract and discussion section to "number" to make our conclusion more rigorous.**

3) Data obtained from the proteomic approach appear under-discussed. In particular, the observed up-regulation of key proteins of the tricarboxylic acid cycle seems to suggest a SM-induced energy shift from glycolysis to oxidative phosphorylation in the hippocampus of T rats. This, together with the observed mitochondrial ultrastructural alterations, could suggest a SM-induced mitochondrial damage. From this point of view, under-discussed is the reported up-regulation of antioxidant enzymes such as SOD2 and Peroxiredoxin 3.

**Reply: Thank you for your wonderful comments.** **We agree with you on energy shift and mitochondrial damage induced by SM, and modified the discussion part of the manuscript. Please refer to it. Although the ultrastructural changes of mitochondria and the up regulation of antioxidant enzymes such as SOD2 and peroxidase 3 were observed, there was no evidence that mitochondria were damaged. On the contrary, our results showed that the expression of metabolism related proteins were up-regulated, such as TCA cycle related enzymes, so we believe that tail suspension caused the up regulation of mitochondrial function.**

4) The described structural alterations which population of mitochondria concern? The synaptic mitochondria? This is not clear.

**Reply: The morphological changes of mitochondria come from the neuronal soma.** **We have changed the relevant expression in the manuscript to make it clearer.**

5) In the manuscript test, the authors often talk about “mitochondrial function”, but no data have been furnished to conclude on respiratory properties, oxidative capacity, energy efficiency, ATP levels. They are encouraged to correct the text or adequate the experimental design to support conclusions.

**Reply: In order to make our conclusion more rigorous, we have changed the word “mitochondrial function” in line 305 to “mitochondrial metabolic function”, the “mitochondrial function” in line 344 to “function related proteins”, and the “function of mitochondria” in line 367 to “morphology of mitochondria” in the revised manuscript.**

6) Overall, the discussion is poor and not focalized.

**Reply: We have revised the discussion part of the manuscript according to the comments of the reviewers, and hope to meet your requirements.**

7) As the authors themselves claim, the tail suspension model simulate fluid shift, muscle atrophy, bone loss, likely producing systemic metabolic adaptations. However, in the discussion, this aspect has been completely neglected even only as a framework for the possible effects of the model on the brain and in particular on the hippocampus.

**Reply: In the discussion part, we explain the possible causes of the phenomena observed in this study.**

Minor:

- in the text, some grammar correction is needed (Introduction: line 76, "are" is missing)

**Reply: Thank you. We have corrected this mistake.**

Reviewer #2: In this study, the authors describe changes occurring within the mitochondrial proteome of rat hippocampus by using the tail suspension microgravity simulation model. The work is well organized, however, some concerns should be addressed by the authors.

Minor revisions

Materials and methods section

- Please, detail the “Processing” and “Consensus” workflow nodes used for the raw data processing via Proteome Discoverer.

- Information on the chromatographic gradient used for LC-MS/MS analyses together with tags used for labelling are missing. Please, specify.

- Does the Uniprot_Rat database include TrEMBL entries? Please, specify by including the version of the UniProt release.

**Reply: Thank you. We have described these methods in detail. Please refer to the TMT- based proteomics analysis section of the revised materials and methods.**

Results section

- In Table 1 and Table 3, please include the Accession Code of the identified proteins.

- In the PPI network, mark with a different colour additional interactors, besides DE identified proteins, if included.

**Reply: We have added Accession Code of the identified proteins to Table1 and Table 3. In addition to the DE identified protein, there is another protein involved in the second shell of interactors, which we have shown in the PPI network diagram with white colour.**

Major revisions

Materials and methods section

The accurate sample quantification is a prerequisite for TMT labelling. Sample up- or under-estimation could tremendously affect the result of the analyses in terms of protein modulation. BCA assay is strongly recommended by ThermoFisher as detailed in the TMT datasheet. Since that it is not specified in the manuscript, did you perform it for samples quantification?

**Reply: Thank you. The protein concentration was quantified by BCA assay and described in the method section of the revised manuscript.**

Discussion

The discussion of proteomic data is very poor. Moreover, besides indicating the pathways affected by changes in protein expression, the authors don’t provide data concerning neither the function nor the morphology of mitochondria.

Please, better focus the proteomic data discussion. In addition, attenuate the sentences concerning functional analyses if any additional experimental data are provided.

**Reply: Thank you for your comments. We supplemented the data of mitochondrial morphological analysis in the revised manuscript, and changed the word “mitochondrial function” to “mitochondrial number” in appropriate places; The discussion part has been improved to focus more on proteomic data.**

6. PLOS authors have the option to publish the peer review history of their article (what does this mean?). If published, this will include your full peer review and any attached files.

If you choose “no”, your identity will remain anonymous but your review may still be made public.

Do you want your identity to be public for this peer review? For information about this choice, including consent withdrawal, please see our Privacy Policy.

Reviewer #1: Yes: Elena Silvestri

Reviewer #2: No

[NOTE: If reviewer comments were submitted as an attachment file, they will be attached to this email and accessible via the submission site. Please log into your account, locate the manuscript record, and check for the action link "View Attachments". If this link does not appear, there are no attachment files.]

While revising your submission, please upload your figure files to the Preflight Analysis and Conversion Engine (PACE) digital diagnostic tool, https://pacev2.apexcovantage.com/. PACE helps ensure that figures meet PLOS requirements. To use PACE, you must first register as a user. Registration is free. Then, login and navigate to the UPLOAD tab, where you will find detailed instructions on how to use the tool. If you encounter any issues or have any questions when using PACE, please email PLOS at figures@plos.org. Please note that Supporting Information files do not need this step.

In compliance with data protection regulations, you may request that we remove your personal registration details at any time. (Remove my information/details). Please contact the publication office if you have any questions.
